# The impact of transformational leadership on the workplace well-being of employees with disabilities: series mediation and moderation process

**DOI:** 10.3389/fpubh.2025.1506257

**Published:** 2025-05-15

**Authors:** Abdulaziz Alfalih, Wided Ragmoun

**Affiliations:** Department of Business Administration, College of Business and Economics, Qassim University, Buraydah, Saudi Arabia

**Keywords:** employees with disabilities, assistive technology, transformational leadership, organizational factors, personal factors, culture, disability technology

## Abstract

This paper investigates the impact of transformational leadership on workplace well-being for employees with disabilities through the integration of assistive technology (AT) and its influencing factors. Despite the growing recognition of the importance of inclusivity in the workplace, employees with disabilities frequently encounter barriers that can hinder their well-being and participation. This research examines how transformational leadership can shape the organizational context through the adoption, utilization, and effectiveness of AT in promoting employee well-being while also considering the impact of psychological factors. A questionnaire was employed to collect responses from 432 employees with disabilities. Based on the gathered data, Structural Equation Modeling (SEM) with SmartPLS was utilized to test the research hypotheses and produce results. The findings reveal that transformational leadership fosters a positive organizational context by nurturing a culture of inclusion and support with *β* = 0.081. This context promotes integrating assistive technology to enhance the well-being of disabled employees in the workplace. Additionally, the results confirm that transformational leadership encourages the development of psychological factors such as self-confidence and self-efficacy as key determinants of well-being, with *β* = 0.435. This paper contributes to the existing literature by providing a nuanced understanding of the relationship between transformational leadership, assistive technology, and the well-being of employees with disabilities. It offers comprehensive practical insights for organizations seeking to improve their inclusive practices and support employees with disabilities in achieving their full work potential.

## 1 Introduction

The workplace environment is crucial in shaping all employees’ well-being and job satisfaction (1), including those with disabilities. However, individuals with disabilities often encounter unique challenges that affect their productivity, engagement, and overall work experience (2, 3). These challenges may include physical barriers, communication difficulties, cognitive limitations, and restricted access to workplace accommodations (121). Despite these obstacles, employees with disabilities have demonstrated their ability to contribute significantly to organizations when provided with the necessary tools, support, and inclusive practices leadership (4).

Transformational leadership has received significant attention for its positive impact on employee motivation, job satisfaction, and overall well-being (5). Transformational leaders inspire and motivate employees by promoting innovation, cultivating a sense of purpose, and offering personalized support (6, 7). For employees with disabilities, such leadership can be particularly crucial, as it fosters an environment that is more accommodating, inclusive, and responsive to their specific needs. The focus of transformational leadership on individualized consideration enables leaders to tackle the diverse challenges faced by employees with disabilities, ensuring they have access to the resources, tools, and opportunities required to flourish in the workplace (8, 9).

In recent years, the adoption of Assistive Technology (AT) has been recognized as a crucial enabler for employees with disabilities (10, 11). AT includes various tools and devices to help individuals overcome physical, sensory, or cognitive challenges when performing job tasks (12). These technologies can range from simple items like ergonomic desks and adaptive keyboards to more advanced solutions such as speech recognition software, screen readers, and hearing aids (13). When effectively integrated into the workplace, AT can significantly improve the work performance, independence, and well-being of employees with disabilities. However, the success of AT in enhancing workplace outcomes often relies on organizational support (14), leadership practices (15), employee engagement (16), and organizational culture (17).

Under specific conditions, the intersection of transformational leadership and assistive technology offers a unique opportunity to enhance workplace well-being for employees with disabilities. Transformational leaders can play a vital role in creating an environment where employees feel empowered to utilize assistive technology effectively (18, 19), resulting in improved productivity and well-being. By providing personalized support and promoting technology that meets employees’ needs, transformational leaders can help reduce the stigma often associated with disability, ensuring that employees feel valued and included.

While there is growing recognition of transformational leadership and assistive technology in improving workplace outcomes for employees with disabilities, a limited understanding remains about how these factors interact to influence overall well-being.

Previous research has primarily focused on leadership’s impact on overall employee well-being (20–22) or the benefits of assistive technology (AT) in enhancing performance (23–25). However, there have been few studies examining how transformational leadership practices can facilitate the adoption and effective use of AT to improve workplace well-being for employees with disabilities. This research gap is especially significant considering the increasing emphasis on diversity and inclusion in contemporary organizations.

Despite increased awareness of the significance of leadership and technology in improving the workplace experience for employees with disabilities, a substantial gap still exists in understanding how transformational leadership and assistive technology interact to affect employee well-being. Previous studies have examined these variables individually, but limited research has explored the combined impact of transformational leadership and assistive technology on workplace outcomes for employees with disabilities, as well as the other factors that may influence this interaction.

This paper aims to address this gap by examining the impact of transformational leadership on the workplace well-being of employees with disabilities through the lens of assistive technology integration. We suppose that this effect can be mediated and moderated by various factors, including organizational and psychological elements. By exploring the relationship between leadership behaviors, technology use and adoption, and employee well-being under specific conditions, this study will contribute to a deeper understanding of how organizations can create more inclusive and supportive environments for employees with disabilities. The research will also investigate the mediating role of assistive technology integration and organizational context, analyzing how its adoption and effective use are influenced by transformational leadership, culture, and organizational factors and how, in turn, these elements affect the psychological and social well-being of employees with disabilities.

Ultimately, this research aims to provide valuable insights for organizational leaders on utilizing transformational leadership practices and assistive technologies to enhance the workplace experience for employees with disabilities, fostering more inclusive and effective environments. By examining this mediation effect, the paper contributes to the broader body of knowledge regarding the role of leadership styles in enhancing employee well-being. It expands existing leadership theories by introducing the unique context of disabled employees, which may differ from the general workforce in terms of workplace dynamics, social integration, and psychological needs. Furthermore, the study connects leadership theories with employee well-being models, particularly within a marginalized or unique employee group (disabled employees). It offers insights into how leadership practices influence performance outcomes and affect the mental health, social integration, and overall well-being of employees with disabilities. The research presents a multi-dimensional approach by accounting for organizational, personal, and cultural factors. This enhances our understanding of how different contexts shape the relationship between leadership and well-being, thereby broadening theoretical frameworks that may have previously overlooked these influencing factors.

This study adopts a pragmatic approach and offers practical insights for organizations on how transformative leadership can enhance the well-being of employees with disabilities. This may include recommendations regarding leadership training programs, organizational culture, and policies that promote inclusive leadership styles. The findings can guide the development of organizational policies aimed explicitly at disability inclusion, mental health, and employee engagement. Organizations can leverage this research to create leadership programs that foster empathy, support, and motivation among leaders, ensuring that employees with disabilities feel valued, respected, and included.

This paper is structured into four main sections. The first section includes a literature review, which identifies and defines all the research concepts while aiming to derive the research hypothesis. The second section outlines the methodology related to the population and the techniques used. The third section presents and analyzes the results, providing new evidence that supports our theoretical approach. The final section summarizes the conclusions, contributions, future research directions, and limitations.

## 2 Literature review and hypothesis

In this part, the main theories used to assess and structure various interrelationships between variables, including mediating and moderating effects, are presented and detailed. The literature review is then discussed to establish the hypothesis and its theoretical references.

### 2.1 Theories

#### 2.1.1 Job demand—resource theory (JD-R)

It is a framework that examines the balance between job demands in terms of physical and psychological aspects and job resources to achieve work goals and stimulate employee growth (26). This theory is based on two processes to achieve this balance, influencing employee well-being and performance. The first is the health impairment process, where high demands and low resources lead to strain, and the motivational process, where high resources and low demands stimulate individual performance and engagement (27). Using this theory, Demerouti and Bakker (28) demonstrated that the interaction between family, personal, and organizational demands and resource use predicts outcomes during the crisis.

It is also acknowledged that this theory supports the importance of managing job demands and resources to enhance individual well-being and job performance (29). In this research, transformational leadership will oversee organizational context and psychological factors to determine the well-being of disabled employees. Furthermore, this theory suggests that the balance between demands and resources influences work performance through engagement, which represents, in turn, one of the ultimate objectives of transformational leadership as a leadership style.

To conclude, this theory emphasizes the importance of a balance between demand and resources in jobs on one side, and the significance of both job characteristics and personal resources in influencing employee outcomes on the other side. It helps explain how and why transformational leadership can stimulate well-being as an outcome, and how some factors can regulate this effect based on their nature.

#### 2.1.2 Technology acceptance model (TAM)

TAM is an important framework that helps to understand how individuals accept and utilize technology based on perceived ease of use and perceived usefulness, which determine the final intention to use (30, 31). This model was revisited to emphasize the role of Attitude Toward Using in enhancing its predictive power, particularly in educational contexts. The Technology Acceptance Model (TAM) explains how users accept and use technology, focusing on perceived usefulness and ease of use. It aids marketers in designing strategies to enhance consumer adoption and usage of technological products and services.

It examines how technology is accepted based on its perceived usefulness and ease of use, which determines user attitudes and the adoption of new technologies. Many studies have previously used this theory in the learning process involving technology and its integration (9, 32). Another important aspect of this framework that seems particularly useful in this research is the significance of cognitive perception in influencing technology use.

A brief analysis of this theory enables us to consider it when structuring the impact of assistive technology to stimulate and enhance the generation of well-being through leadership, organization, and psychological factors. The integration of factors that determine TAM can influence the level of well-being and the relative importance of other factors affecting this level.

### 2.2 Hypothesis

#### 2.2.1 Transformational leadership and organizational context

Transformational leadership has received considerable attention in organizational research because of its ability to foster innovation (33, 34), enhance employee engagement (35, 36), and drive overall organizational success. This leadership style, defined by idealized influence, inspirational motivation, intellectual stimulation, and individualized consideration (37), is widely acknowledged for its positive effect on the organizational environment (38, 39). Research indicates that transformational leadership positively influences organizational performance (23). Nasir et al. (40) suggest that transformational leaders inspire employees to exceed expectations, increasing productivity and innovation. Qalati et al. (41) conducted a meta-analysis demonstrating that transformational leadership is strongly associated with improved employee performance and organizational effectiveness. Such leaders create a compelling vision that aligns organizational goals with employees’ intrinsic motivation, fostering a more committed workforce (42).

Transformational leadership is also linked to higher levels of employee engagement and job satisfaction. Research indicates that employees who work under transformational leaders experience increased motivation, autonomy, and opportunities for personal development (43, 44). A study by Krishna et al. (45) showed that transformational leadership fosters a positive work environment, improving job satisfaction and decreasing turnover rates. This leadership style cultivates trust and empowerment, further reinforcing its impact on enhancing organizational culture (46). Transformational leadership significantly influences organizational culture by fostering shared values and a unified mission (47, 48). Studies by Dinc et al. (49) indicate that transformational leadership enhances organizational commitment through the promotion of a sense of purpose and belonging. Employees feel more connected to the organization’s goals, resulting in increased loyalty and diminished resistance to change (50).

*H1*: TL is related positively to the organizational context

#### 2.2.2 Organizational context and assistive technology (AT) integration

Assistive technology (AT) enhances accessibility, productivity, and inclusion for individuals with disabilities (51, 52). However, successfully integrating AT relies on several factors derived from the technology acceptance theory (53). The literature review identified critical factors, such as the organizational context, which includes leadership support, policies, training, and culture, that positively influence the adoption and integration of AT.

Organizational policies that support infrastructure are crucial in facilitating AT adoption (54, 55). Institutions with well-defined accessibility policies and dedicated support structures tend to see better integration outcomes. Mahmoudi-Dehaki et al. (24) highlights that regulatory compliance and structured policies promote the use of AT in educational and workplace settings. In the same vein, Karki et al. (56) discuss how accessibility policies can create an inclusive work culture, enhancing the effectiveness of AT solutions.

An inclusive and technology-friendly organizational culture significantly influences AT integration (57). Chaudhry et al. (58) found that organizations with strong diversity and inclusion initiatives report greater AT acceptance. Additionally, Marinaci et al. (35) emphasize that peer support and positive workplace attitudes improve AT sustainability.

*H2*: Organizational context has a positive effect on AT integration.

#### 2.2.3 Assistive technology (AT) integration and the well-being of disabled employees

Assistive Technology (AT) is crucial for enhancing workplace accessibility and inclusion for employees with disabilities (59). AT encompasses various tools, including screen readers, speech recognition software, ergonomic office equipment, and mobility aids, improving job performance, psychological well-being, and overall quality of work life (60). Models such as the Social Model of Disability support the integration of AT in the workplace (61). This model emphasizes removing environmental barriers to enable full participation (62). Additionally, Koroglu and Ozmen (63) demonstrated that AT can act as a job resource that mitigates work stressors and enhances employee engagement by adopting the Job Demands-Resources (JD-R) model.

Research consistently shows that assistive technology (AT) improves job retention, productivity, and satisfaction among employees with disabilities (10, 64). Most studies indicate that employees who receive appropriate AT accommodations face fewer workplace challenges and are better able to meet job expectations (65, 66). Employees’ psychological well-being is closely linked to their ability to perform job tasks independently. Several studies have shown that using assistive technology (AT) reduces anxiety and stress related to workplace barriers (67). Also, Marinaci et al., (59) found that AT users reported higher levels of workplace confidence and job satisfaction compared to those who did not have access to such technologies.

Additionally, AT promotes greater social inclusion by enabling employees to communicate and collaborate effectively with their colleagues (52). For example, digital communication tools allow employees with hearing or speech impairments to engage more fully in workplace interactions, thereby fostering a more inclusive organizational culture (64).

*H3*: AT integration has a positive effect on the well-being of disabled employees

#### 2.2.4 Transformational leadership and the well-being of disabled employees

Transformational leadership is widely recognized as an influential style that boosts employee motivation, job satisfaction, and overall well-being (68). This approach, characterized by vision, inspiration, individualized consideration, and intellectual stimulation, is linked to various positive outcomes in the workplace.

To assess this effect, two theories can be applied: transformational leadership theory posits that leaders who inspire, challenge, and support their employees can significantly enhance job engagement and psychological well-being (43). Additionally, the Job Demands-Resources (JD-R) model suggests that transformational leadership acts as a job resource that reduces stressors and promotes a positive work environment, particularly for employees with disabilities who may face additional challenges in the workplace (69). Studies indicate that transformational leaders provide emotional support and foster inclusive workplace cultures, which are particularly advantageous for employees with disabilities (70). For example, individualized consideration, a vital component of transformational leadership, ensures that leaders recognize and address the specific needs of disabled employees, thereby improving their work experience (71).

Transformational leadership is linked to lower stress and burnout, crucial issues for disabled employees who often encounter workplace barriers (72, 121). Studies indicate that employees under transformational leaders report higher self-efficacy, resilience, and job satisfaction (73, 74). In their research, Song et al. (75) showed that employees with supportive transformational leaders experience less psychological distress and a stronger sense of belonging within their organizations. A key advantage of transformational leadership is its ability to foster an inclusive work environment. Research by Carrington et al. (70) revealed that transformational leaders actively encourage diversity and ensure that disabled employees feel valued and included.

*H4*: Transformational leadership is related positively to the well-being of disabled employees.

#### 2.2.5 The moderating role of psychological factors in the impact of transformational leadership on the well-being of disabled employees

Transformational Leadership (TL) is widely acknowledged as a powerful leadership style that fosters motivation, empowerment, and workplace well-being (76). However, its effects on disabled employees vary and may be influenced by different psychological factors, such as resilience, self-efficacy, and perceived organizational support (77–79). TL is built on four core dimensions: idealized influence, inspirational motivation, intellectual stimulation, and individualized consideration (80). According to Afshari (81), idealized influence relies on psychological factors that can either promote or hinder acceptance. TL has been linked to increased job satisfaction and mental well-being (5). Psychological factors can affect this relationship, acting as either buffers or amplifiers in shaping employee well-being (82, 83).

A study by Mofti et al. (84) found that transformational leadership (TL) was positively associated with employee well-being; however, the strength of this relationship depended on employees’ levels of psychological resilience. Similarly, Wong (85) demonstrated that self-efficacy significantly moderated the relationship between TL and work engagement among employees with disabilities. Another study by Kusi et al. (86) indicated that perceived organizational support (POS) strengthened the positive effects of TL on job satisfaction and mental health. This research selected three main psychological factors that seem to be directly related to the TL and its effects: resilience, self-efficacy, and perceived organizational support. This selection is due to the recurrent aspects of these variables in most previous studies.

Resilience, defined as adapting and recovering from adversity (87), is crucial in how disabled employees respond to TL. Research suggests that employees with higher resilience experience more significant benefits from TL, as they can better navigate workplace challenges and leverage leadership support (88).

Self-efficacy, the belief in one’s ability to achieve goals (89), is another essential moderator. Employees with high self-efficacy tend to view transformational leadership (TL) behaviors as more empowering and enhance their well-being. Conversely, those with low self-efficacy may find it difficult to fully benefit from TL, as they often experience self-doubt and workplace pressures (90).

Perceived organizational support refers to employees’ beliefs about the extent to which their organization values their contributions and well-being (91). Research shows that when employees with disabilities perceive high levels of support, the positive effects of transformational leadership on their well-being are amplified (92).

*H5*: Psychological factors moderate the impact of TL on the well-being of disabled employees.

**Figure 1:**
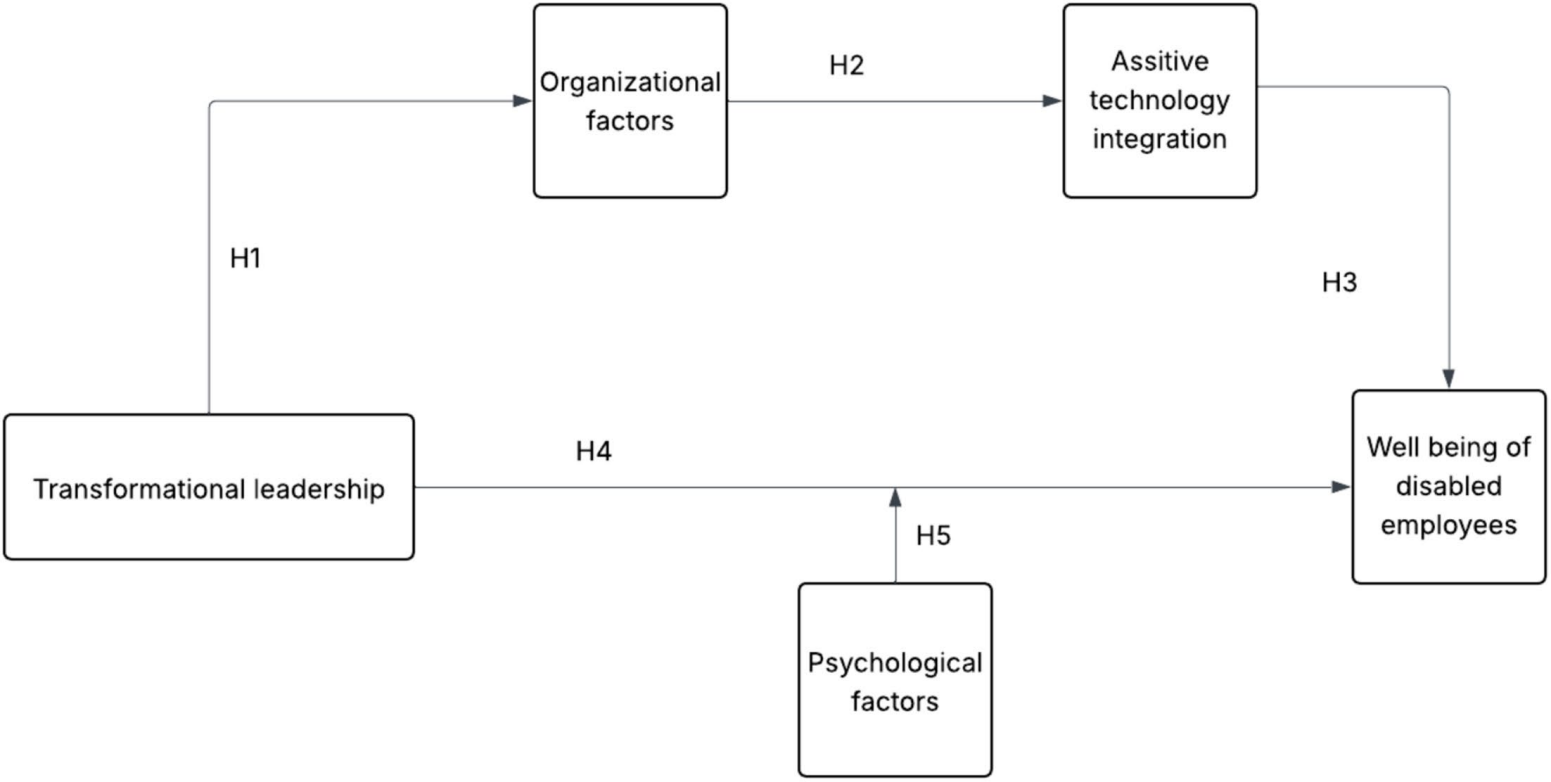
Theorical framework.

Figure 1 illustrates the theoretical model defined by the authors, which includes five main variables and five hypotheses: three related to the series of mediation and one concerning the moderating effect.

As shown in Figure 1, the five key concepts interact to determine well-being as a psychological state based on transformational leadership as an organizational practice. Table 1 presents clear definitions of each concept and the corresponding references to enhance the understanding of different concepts and highlight how and why these interactions are significant. Only the most relevant definitions are mentioned.

**Table 1:** Research concepts and definitions.

Concepts	Definitions	References
Transformational leadership (TL)	TL is a process in which leader aims to stimulate employees by inspiring, energizing and supporting them to guarantee a high level of commitment and trust. TL play a critical role in defining and creating supportive environments for marginalized groups by fostering the use of assistive technologies, promoting inclusivity developing a culture based on psychological safety. TL contributes to enhance well-being at work by stimulating psychological empowerment, promoting positive attitudes and supporting a supportive affective state.	Yuan et al. (102) Shields and Hesbol (103) Gyu Park et al. (104)
Psychological factors (PF)	Psychological factors represent an internal process and characteristics that determine individuals perception and response towards the environment. Some studies consider these factors as personal resources related to some inner capacities used to manage stress, increase persona performance and improve psychological resilience in work place. It is a multidimensional concept that integrate some psychological mechanisms such as: self-efficacy, resilience, optimism and psychological capital.	D’Amato and Zijlstra (105) Chaudhry et al. (106) Engelmann et al. (107)
Organizational context (OC)	OC represents situational opportunities and constraints that determine organizational behavior and different functional relationships between employees and organizational levels. It identifies situational factors, structures, and cultural elements that can affect behaviors in internal and external levels.	Banwo et al. (108) Lam et al. (109)
Assistive technology integration	Tools or systems that assist and aid persons with disabilities to perform job effectively. It eliminates and reduce barriers in term of physical access and communication AT can be equipment, item, software program, or a system that improve or maintain functional capabilities of people with disabilities in work	Maalim and MacLachlan (51) Ding et al. (110)
Well-being (WB)	Well-being at work reflect the quality of work functioning and employee’s experience in terms of physical health, state, emotions, relationships, and job satisfaction. It is directly related to the emotional state and feeling. It is the balance between resource pool challenges faced by persons during their life or work.	Hameli et al. (111) Yoon et al. (112)

## 3 Methodology

### 3.1 Procedure

The data collection process was developed through a questionnaire administered to disabled employees in industrial and service organizations across various regions of Saudi Arabia, particularly Qassim, Riyadh, and Jeddah. The questionnaire was initially written in English and later translated into Arabic under the supervision of experts. It was then distributed via Google Forms, addressed to a supervisor, or, where possible, directly to the employees. Ample time was given to the respondents to return their answers to the research team. Throughout this process, we aimed to maximize the response rate while ensuring that all data would remain anonymous and be used solely for research purposes. Five hundred questionnaires were distributed, with 432 returned, indicating a response rate of about 86.4%. In this research, 67% were male and 33% were female. The corresponding age was ranged between 23 and 40. Nearly (45%) of employees have experience of one to 5 years in the organization.

Variables and dimensions were appreciated using a 5-point Likert scale (from 1 to 5) for items used, with 1 (strongly disagree) and 5 (strongly agree), except for one dealing with TL, which ranged from never to always (see Table 2).

**Table 2:** Details each variable, the number of dimensions, their corresponding items, and references used.

Variables	Dimensions	Number of items	References
Transformational leadership	Idealized influence Inspirational motivation Intellectual stimulation Individualized consideration	12	Bass et al. (113) Avolio et al. (114)
Organizational context	Organizational cultures Organizational structure Communication and decision making	12	Porter and Mclaughlin (115) Doolen et al. (116)
Psychological factors	Self efficacy Resilience Stress and coping	9	Hajek et al. (117)
Assistive technology integration	Availability of assistive technology Usage and accessibility Effectiveness of assistive technology Acceptance of assistive technology	12	López-Fernández et al. (118)
Well-being	The Job Satisfaction and Well-being Scale (JSWS) The Psychological Well-being Scale (PWB)	8	Judge and Watanabe (119) Ryff (120)

During the explorative approach, Cronbach’s alpha was used to assess the reliability of each dimension. The standard deviation and test of correlations for all variables were also calculated. Table 3 shows different values.

**Table 3:** Descriptive statistics amongst research variables (*N* = 432).

Variable	M	SD	1	2	3	4	5
Transformational leadership	5.31	0.79	(0.81)				
Organizational context	3.71	0.69	0.541 **	(0.85)			
AT integration	4.16	0.89	0.347 **	0.283 **	(0.92)		
Psychological factors	3.45	0.92	0.385 *	0.626**	0.641 **	(0.93)	
Well-being	4.14	0.90	0.412 **	0.392 **	0.347 **	0.445 **	(0.89)

*p < 0.05, **p < 0.01, numbers in parentheses are Cronbach’s alphas.

### 3.2 Confirmatory factor analysis (CFA)

We utilized Smart–PLS for confirmatory factor analysis to check the reliability and validity of all research variables and the test for common method bias (93). In addition, alternate models were tested for five factors on the theoretical model to provide the model fit according to the recommendations of Preacher and Hayes (94). Corresponding results are detailed in Table 4. We have to remember that, in this state, there is no issue with discriminant validity.

**Table 4:** Comparison of measurement models.

Model–variables	χ^2^	*df*	GFI	IFI	CFI	TLI	RMSEA
Five factors	523.17***	309	0.91	0.94	0.94	0.90	0.039
Four-factor model (AT integration and well-being into one factor)	634.56 ***	317	0.80	0.91	0.92	0.89	0.089
Three-factor model (organizational factors, transformational leadership, and AT integration into one factor)			0.76	0.79	0.82	0.80	0.071
Two-factor model (organizational factors, Transformational Leadership, psychological factors, and well-being into one factor)	831.24 ***	324	0.82	0.87	0.81	0.85	0.089
One factor model	1418.34***	338	0.82	0.85	0.85	0.79	0.116

*N* = 432 ***p < 0.001. RMSEA, root mean square error of approximation. TLI, Tucker–Lewis index. CFI, comparative fit index. GFI, goodness fit index. IFI, incremental fit index.

The analysis confirmed that the 5-factor model (AT integration, transformational leadership, organizational factors, psychological factors, and well-being) fit well (x^2^ = 523.17, (RMSEA) = 0.039, (GFI) = 0.91, (IFI) = 0.94, (CFI) = 0.94, (TLI) = 0.90), and it was remarkably higher than the 1-factor model (x^2^ = 1418.34, RMSEA = 0.116, GFI = 0.82, IFI = 0.85, CFI = 0.85, TLI = 0.79). Based on these results, the standard method bias for this model’s research is non-significant. Thus, we can support the model’s fit and satisfaction with all structural equation modeling requirements.

### 3.3 Hypotheses testing

#### 3.3.1 Mediating test

This study aimed to examine the influence of transformational leadership (TL) on the well-being of disabled employees at work through organizational factors and assistive technology (AT) integration. The literature review presents a serial mediation hypothesis among these variables. In this context, we used SmartPLS to test the mediating and moderating effects (Table 5), considering TL as an independent variable, organizational factors and AT integration as mediating variables, and well-being as a dependent variable.

**Table 5:** Statistical results of serial mediation analysis for indirect effect of transformational leadership on innovative work behavior through trust and work engagement.

Variable	Model 1: DV = organizational context	Model 2: DV = AT integration	Model 3: DV = well-being
Gender	0.069 (0.049)	0.021 (0.039)	0.128 (0.044)**
Age	0.121 (0.079)	0.179 (0.076)**	0.191 (0.083)*
Experience	0.041 (0.038)	*−*0.022 (0.029)	0.027 (0.031)
Tl	0.552 (0.073) ***	0.349 (0.071)***	0.253 (0.069)**
Organizational context	–	0.256 (0.061)*	0.163 (0.081)*
AT integration	–	–	0.646 (0.086)***
R-square	0.221	0.312	0.398
Indirect effect 1: TL → organizational context → well-being = 0.081 (0.032) Significant Mediation			
Indirect effect 2: TL → AT integration → well-being = 0.243 (0.051) Significant Mediation			
Indirect effect 3: organizational context → AT integration → well-being = 0.178 (0.0382) Significant Serial Mediation			
Indirect effect 3: TL → organizational context → AT integration → well-being = 0.053 (0.012) Significant Serial Mediation			
Total Effect of Transformational leadership on well-being = 0.350 (0.059)			

*p < 0.05, **p < 0.01, ***p < 0.001.

During this process, as suggested by Preacher and Hayes (94) a bootstrapping test with a CI of 95% was elaborated. Results revealed that TL is related positively to organizational context (*β* = 0.552, *p* < 0.000), which permits the support of H1. Organizational context is significantly and positively associated with AT integration (*β* = 0.256, *p* < 0.05), confirming H2. The same case for AT integration and well-being are positively related (*β* = 0.646, *p* < 0.000). This implies that H3 is supported, too. The findings revealed a positive impact of TL on well-being, which also seems significant (*β* = 0.253, *p* < 0.01). So, H4 is confirmed.

Additionally, statistical results confirmed, according to H5, the mediating effect of organizational context between TL and well-being (*β* = 0.081). At the same level, the indirect impact of TL on well-being through AT integration was significant with (*β* = 0.243), and hypothesis H6 was accepted. The last step related to the serial mediation effect was also accepted, and this effect between TL, organizational context, AT integration, and well-being was supported by all statistical results (*β* = 0.053).

#### 3.3.2 Moderation results

The results regarding the hierarchical moderation regression presented in Table 6 confirmed H5 and supported the moderating effect of psychological factors. The interaction term (TL x psychological factors) was positive and significant (*β* = 0.213, *p* < 0.01). The corresponding plot indicates that the relationship between TL and well-being strengthens as psychological factors increase.

**Table 6:** Moderation analysis for innovative work behavior.

Variable	Model 1	Model 2	Model 3
Age	0.112 (0.085)	0.160 (0.075) **	0.173 (0.081)*
Gender	0.053 (0.048)	0.021 (0.052)	*−*0.109 (0.049)**
Experience	0.031 (0.0278)	*−*0.023 (0.025)	0.021(0.031)
Tl		0.190 (0.0445)**	0.158 (0.069)*
Psychological factors		0.321 (0.045)***	0.289 (0.061)***
TL x psychological factors			0.213 (0.066)**
R-square	0.209	0.278	0.435

*p < 0.05, **p < 0.01, ***p < 0.01.

## 4 Discussion and theoretical contribution

The key objective of this study was to explain, measure, and analyze the synergy between the TL, organizational factors, psychological factors, AT integration, and well-being. Furthermore, we defined a model based on a serial mediating effect and a moderating effect to explain how these variables interact to enhance the well-being of disabled employees in the Saudian organizational context. The results confirmed that the TL engenders higher well-being among disabled employees through AT integration and organizational context. This research increases the understanding of such hierarchical effects to stimulate and maintain the well-being of disabled employees as much as possible.

The findings provide empirical evidence for the importance of TL and its significant role in building employees’ well-being. They confirm this leadership style’s importance and critical aspect, as supported by many previous studies (95–98). Organizational context, in terms of cultures and policies, significantly impacts well-being. Mainly, TL generates a positive context for creativity and collaboration that can maintain the well-being of disabled employees. This is consistent with many previous studies in the field, such as (79, 99, 121).

The significant and positive serial mediating effect of organizational factors and assistive technology (AT) integration indicates that well-being is context-dependent, and simply implementing assistive technology is insufficient to enhance and sustain workplace well-being. Based on the findings, adopting a transformational leadership (TL) approach can help employees cultivate a positive attitude, thereby increasing their job satisfaction and, subsequently, their overall well-being. On the other hand, this leadership method can foster a favorable organizational context by promoting a new cultural perspective and developing supportive organizational policies. By utilizing assistive technology, employees will find it easier to perform their tasks. Consequently, employee well-being in the workplace is enhanced.

Leadership characteristics significantly affect employee well-being by enhancing their self-confidence and motivation, encouraging them to engage in their work.

Therefore, employees seem more motivated and determined under transformational leadership, demonstrating incredible dedication to their work (100). TL engages and inspires employees to enhance their development and increase their efforts for organizational success (121). He strongly emphasizes teamwork, fosters open communication, and encourages innovation (6, 7), generating a harmonious organizational context.

Moreover, TL encourages innovative work behavior among employees. As the existing literature demonstrates (7), this leader can influence employees to become more committed to facilitating innovation and creative behavior. Under these circumstances, the organizational context seems more attractive for good behaviors and well-being.

Engaging employees in various learning activities by TL will enable them to develop innovative solutions and address all workplace issues. This state guarantees well-being, and a new organizational culture rooted in teamwork, collaboration, and happiness is fostered.

This research investigates the impact of assistive technology integration on well-being. It expands the opportunities for technology use in the workplace, particularly for disabled employees, fostering a willingness to use technology in various ways. The integration of assistive technology can facilitate work, direct efforts, and reinforce employees’ confidence in their ability to be independent and productive.

These results align with previous studies on the use of assistive technology in the workplace (24, 101). To promote employee well-being, organizations should strive to create and sustain a positive organizational environment grounded in a collaborative and innovative culture. This effort should be supported by policies that facilitate the integration of assistive technology, which empowers disabled employees to enhance their self-confidence and determination in various ways and at different levels. Transformational leaders possess a broader vision that inspires innovation among their followers individually, ultimately enhancing organizational innovation.

The findings also suggest that psychological factors influence the relationship between transformational leadership (TL) and employee well-being. TL aims to foster self-confidence, resilience, and independence in its followers, enabling them to become more assertive, build self-efficacy, and maintain a positive perception of organizational support. Previous empirical studies back this effect, demonstrating that TL enhances employees’ resilience by establishing a shared objective and a clear vision. Followers feel valued, engaged, and more comfortable making decisions rather than simply following orders. This sense of empowerment is increasingly vital for sensitive disabled employees, for whom self-image and others’ perceptions are significant importance.

## 5 Conclusion, limitations, and futures research

This research underscores the vital role of transformational leadership (TL) in promoting the well-being of employees with disabilities in the workplace by integrating assistive technology (AT) and considering the effects of organizational context and psychological factors. The findings reveal that TL creates a supportive and inclusive work environment that encourages adopting and effectively using AT, ultimately enhancing employees’ overall well-being. Leaders who demonstrate individualized consideration, intellectual stimulation, and inspirational motivation are essential for empowering disabled employees and fostering a culture of inclusion. Furthermore, the study highlights that psychological factors such as resilience, self-efficacy, and perceived organizational support influence the relationship between transformational leadership (TL) and employee well-being. These factors determine how TL affects the adoption and effectiveness of assistive technology (AT), thereby shaping the workplace experiences of employees with disabilities. Organizations that prioritize these elements can foster a more supportive environment where employees with disabilities can excel professionally and personally.

### 5.1 Recommendations and policy suggestions

Research findings show that transformational leadership, assistive technology, and well-being are interdependent under specific conditions: organizational context and psychological factors. This conclusion supports the notion that organizations should prioritize, develop, and integrate leadership training that generates and maximizes transformational behaviors. Additionally, policies related to the integration and use of assistive technology must be established for regular maintenance and updating according to an adequate institutional framework, making this technology a primary objective with clear procedures. In turn, protocols related to the inclusive technology assessment can be implemented to maximize its use and success for disabled employees.

Due to the significance of psychological factors in this process for well-being, implementing specific psychological wellness programs, such as coaching and mental health initiatives, can enhance the psychological support system and stimulate various necessary psychological mechanisms. Nonetheless, the most vital recommendation at this level still emphasizes developing specific well-being metrics to assess their levels and make appropriate decisions when necessary. These comprehensive policy actions outline a new instrumental approach for a critical pathway for disabled employees.

### 5.2 Future studies

While this research offers valuable insights, future studies should investigate how specific disabilities interact with psychological and organizational factors in different workplace environments. Furthermore, longitudinal studies can provide deeper insights into the long-term effects of TL and AT adoption on the well-being of employees with disabilities. By embracing inclusive leadership practices and leveraging assistive technologies, organizations can cultivate a healthier, more equitable work environment for employees with disabilities.

## Data Availability

The raw data supporting the conclusions of this article will be made available by the authors, without undue reservation.
